# Root morphogenetic responses to waterlogging stress in plants: from structural reconfiguration to molecular regulatory mechanisms

**DOI:** 10.3389/fpls.2026.1823032

**Published:** 2026-05-28

**Authors:** Shanshan Sha, Chengcheng Zeng, Xuyi Shang, Bingchen Zou, Tingting Sun, Yuju Yang

**Affiliations:** 1 School of Food Engineering, Harbin University, Harbin, China; 2 College of Plant Protection, Jilin Agricultural University, Changchun, China; 3 Ministry of Education Engineering Research Center for Agricultural Microbial Technology School of Life Sciences, Heilongjiang University, Harbin, China; 4 National Sugar Crop Improvement Centre, College of Advanced Agriculture and Ecological Environment, Heilongjiang University, Harbin, China

**Keywords:** hormonal signaling crosstalk, interspecific variation, morphological plasticity, root morphogenesis, waterlogging stress

## Abstract

Climate change is increasing the frequency of extreme rainfall events, making waterlogging a major constraint on crop production. Waterlogging imposes a composite stress on plants by causing rhizosphere hypoxia and promoting the accumulation of toxic reduced compounds. Root morphological plasticity is a central adaptive strategy under these conditions. It relies on the coordinated deployment of four linked modules: adventitious root (AR) formation, aerenchyma development, barrier formation to radial oxygen loss (ROL), and root system architecture (RSA) remodeling. These responses are initiated by ERF-VII-dependent hypoxia sensing and further shaped by ethylene-auxin interactions, ROS/Ca^2+^ signaling, gaseous regulators such as NO and H_2_S, and the capacity for metabolic reprogramming and carbon reallocation. Differences among species and genotypes likely reflect variation in signaling sensitivity, regulatory-network organization, and metabolic efficiency. In this review, we integrate current knowledge across three levels: root morphological modules, their regulatory networks, and rhizosphere constraints. We highlight key leverage points for improving waterlogging tolerance and propose a mechanistic framework to support both crop breeding and field management under increasingly flood-prone conditions.

## 1 Introduction

Against the backdrop of ongoing climate change, the frequency and intensity of extreme precipitation events continue to rise, making waterlogging one of the most consequential abiotic constraints on crop productivity and ecosystem stability (Nikolić et al., 2025). Unlike drought or salinity, which primarily disrupt plant function through osmotic imbalance, waterlogging rapidly reconfigures the rhizosphere gas-diffusion regime: prolonged pore waterlogging sharply reduces the soil oxygen diffusion coefficient, driving the root zone into hypoxia—and, in severe cases, anoxia—within a short time window. The resulting oxygen deficiency not only directly suppresses mitochondrial aerobic respiration, but also coincides with a decline in redox potential, the accumulation of reduced ions and sulfides, and a reactive oxygen species (ROS) burst during subsequent reoxygenation. Together, these processes generate a composite stress environment centered on oxygen limitation yet characterized by metabolic disarray and chemical toxicity (Li et al., 2021).

Roots are the first organ to perceive waterlogging and remain chronically exposed to oxygen deprivation; they therefore constitute the primary interface through which plants build waterlogging tolerance. Accumulating evidence indicates that adaptation to waterlogging cannot be explained by metabolic adjustment or antioxidant defense alone; rather, it crucially depends on rapid, structural-level reprogramming of the root system. This includes adventitious root formation, aerenchyma development, regulation of radial oxygen loss (ROL), and reconfiguration of root system architecture (RSA) (Yamauchi et al., 2018). By rebuilding internal oxygen transport pathways, redistributing absorptive structures in space, and modulating rhizosphere redox status, these morphogenetic transitions help sustain basal root metabolism and nutrient uptake under persistent hypoxia, thereby largely determining survival and post-stress recovery capacity under waterlogging (Liu et al., 2024).

Despite notable advances in dissecting root morphological responses to waterlogging, current research remains fragmented and often reductionist. Most studies focus on a single morphogenetic module (e.g., adventitious roots, aerenchyma, or the ROL barrier) or a single signaling pathway (e.g., ethylene or ROS) (Gu et al., 2024). Critically, mechanistic work has largely emphasized how individual traits are induced, while offering limited, system-level explanations for the coordination and temporal ordering among modules—an omission that severely limits our ability to account for the strikingly different trait combinations assembled by distinct species and genotypes under hypoxia. Meanwhile, although molecular studies have uncovered multiple hormonal and hypoxia-responsive pathways, how these signals are integrated and translated into spatially precise morphogenetic outputs, and how energetic state and carbon supply constrain the magnitude and persistence of such structural remodeling—still lacks a unified mechanistic framework (He W. et al., 2023).

With deepening investigation, waterlogging adaptation is increasingly understood as a structurally executed process with root morphogenesis at its core. Hypoxia perception and hormonal networks provide developmental initiation cues and spatial patterning signals; aeration structures and shifts in root-type composition underpin oxygen acquisition and transport; and, importantly, cellular energy status and carbon allocation capacity determine whether these structures can be efficiently built and durably maintained. Interspecific and genotypic variation in waterlogging tolerance, in essence, reflects how these modules are combinatorially deployed and how well they match the prevailing rhizosphere constraints (Li and Rao, 2024).

In this context, the present review takes “root morphogenetic responses” as the central thread and synthesizes progress across four levels: waterlogging-driven rhizosphere changes, morphogenetic execution modules, cross-scale regulatory networks, and interspecific diversification of adaptive strategies. From functional and temporal perspectives, we systematically delineate the coordination among adventitious rooting, aerenchyma formation, ROL regulation, and root system architectural remodeling. We then integrate hypoxia sensing, hormonal crosstalk, redox signaling, and energy-metabolism coupling into a coherent regulatory framework to clarify the molecular basis of root morphogenesis under waterlogging. Finally, by comparing morphogenetic strategies across species and genotypes, we highlight the species-specific nature of root responses and their underlying molecular determinants, thereby providing a mechanistic foundation to inform breeding for waterlogging-resilient crops. To further guide crop improvement, we also highlight hypoxia tolerance thresholds and effective waterlogging durations in major cereal and legume crops, which provide practical benchmarks for phenotyping and breeding.

## 2 Rhizosphere signatures of waterlogging and mechanisms of composite injury

Waterlogging is not a single-factor stress. Rather, it rapidly generates a rhizosphere environment in which constrained physical diffusion, a shift in redox regime, and reprogrammed microbial processes act together to impose hypoxia, chemical toxicity, and reoxygenation risk. Importantly, this environment is not merely a passive background. It defines the boundary conditions that determine root energetic status, the maintenance of cellular homeostasis, and the feasibility of subsequent morphogenetic remodeling. Accordingly, resolving the temporal dynamics of rhizosphere physical, chemical, and biological processes under waterlogging is a prerequisite for understanding adaptive root structural reconfiguration (Sherwood et al., 2025).

In this review, the term “rhizosphere signatures of waterlogging” refers to the characteristic and dynamically coupled physical, chemical, and biological features that emerge after soil inundation. These include rapid oxygen depletion, a decline in redox potential, accumulation of reduced ions and metabolites, and restructuring of microbial functions. Defining these signatures is important because root morphological adaptation does not occur in isolation; it is built under, and constrained by, this evolving rhizosphere context.

### 2.1 Physical constraints in the rhizosphere: impaired oxygen diffusion and microenvironmental deterioration

Following waterlogging, soil pores remain filled with water, causing a precipitous decline in gas diffusion efficiency. Because oxygen diffusion through water-saturated pore space is markedly impeded relative to air, rhizosphere oxygen supply rapidly becomes insufficient to meet root respiratory demand (Li W. et al., 2025). In most soil types, rhizosphere O_2_ can be depleted within hours as a result of root respiration and microbial oxygen consumption, establishing a stable hypoxic—and sometimes anoxic—microenvironment. Spatially, near-surface layers maintain comparatively higher O_2_ due to exchange with the atmosphere, whereas deeper soil often shifts into a persistently reducing state, generating a pronounced top-down oxygen gradient (Park et al., 2025).

Beyond oxygen limitation *per se*, water saturation fundamentally alters soil physical properties. Impaired pore-gas exchange promotes the accumulation of CO_2_ and other gases in the rhizosphere, while anaerobiosis-associated volatiles can further reshape local gas composition and diffusion conditions. In parallel, increased soil bulk density, pore-structure collapse, and higher mechanical impedance directly suppress root elongation and root hair formation. Collectively, these changes not only determine the intensity of hypoxia but also spatially delimit where root morphogenetic remodeling can occur (Wang Z. et al., 2025).

Notably, many experimental studies still use waterlogging duration as the primary descriptor of stress severity. This is often insufficient. Soil texture, pore structure, and initial water status strongly affect oxygen diffusion, so the same exposure time can impose very different oxygen burdens across systems. A more robust framework is to interpret duration together with species- or genotype-specific hypoxia thresholds, because the onset of structural and metabolic injury depends not only on how long roots remain submerged, but also on how quickly root-zone oxygen falls below the tolerance range of a given plant material (Yu et al., 2022). Accordingly, oxygen partial pressure, diffusion metrics, and redox potential (Eh) should be incorporated as baseline descriptors in mechanistic studies and phenotyping pipelines.

### 2.2 Chemical upheaval in the rhizosphere: redox disequilibrium and toxin accumulation

Rhizosphere hypoxia first precipitates a systemic shift in redox conditions. After waterlogging, soil Eh declines rapidly, transitioning from positive values typical of aerobic soils to a reducing regime, thereby driving a cascade of chemical transformations characterized by sequential replacement of terminal electron acceptors. Oxidized ions such as Fe^3+^ and Mn^4+^ are reduced to Fe^2+^ and Mn^2+^, sulfate reduction and methanogenesis intensify, and organic acids and other reduced metabolites may progressively accumulate in the rhizosphere (Wang Z. et al., 2019).

These changes do not merely reconfigure nutrient speciation, they directly constitute chemical stressors for roots. Excess Fe^2+^ and Mn^2+^ can compromise membrane stability and disrupt ionic balance. Sulfides can inhibit mitochondrial cytochrome c oxidase, further blocking the respiratory chain and exacerbating the energetic crisis (Tian et al., 2023; Chen et al., 2024b). Accumulated organic acids may shift rhizosphere pH and aggravate membrane permeability damage (Luo et al., 2023). Therefore, rhizosphere chemical transformation and root energy failure are not separable phenomena, they reinforce one another through convergent effects on respiration inhibition, ion disequilibrium, and membrane injury.

Moreover, upon drainage and reoxygenation, rapid oxidation of reduced compounds coincides with restoration of mitochondrial electron transport, frequently provoking sharp ROS accumulation and a “reoxygenation oxidative shock”. The magnitude of this oxidative burst can largely determine whether tissue damage remains reversible (He N. et al., 2023). This reoxygenation-phase risk is therefore itself a critical constraint on morphological recovery.

### 2.3 Rewiring of rhizosphere biological processes: establishment of anaerobic metabolic networks and their bidirectional effects

The low-oxygen environment imposed by waterlogging inevitably triggers profound restructuring of rhizosphere microbial processes. Community composition typically shifts away from strict aerobes, whereas facultative anaerobes and anaerobiosis-associated functions are enriched, fermentation, sulfate reduction, and methanogenesis emerge as prominent metabolic routes (Maciag and Krzyżanowska, 2025). This transition is not stochastic. Instead, it reflects niche filtering driven by electron-acceptor availability and progressively consolidates a new network of metabolic interactions (Dey et al., 2025).

Microbial restructuring can affect plants in clearly dualistic ways. On one hand, microbial metabolites such as sulfides and organic acids can amplify rhizosphere toxicity and accelerate root injury. On the other hand, some studies suggest that, under specific contexts, rhizospheres may become enriched in growth-promoting or stress-mitigating taxa, for example, ACC deaminase-carrying strains can modulate ethylene precursor levels and thereby partially alleviate stress-associated growth inhibition under certain conditions (Chaudhary et al., 2022).

Nevertheless, most current evidence for microbiome shifts remains largely correlational. Whether key functional groups causally contribute to waterlogging tolerance formation is still insufficiently validated in controlled systems. Moving forward, integrating microbial processes into a root morphogenetic adaptation framework will require axenic platforms, synthetic communities, and targeted re-inoculation designs to establish causal links among microbial function, rhizosphere redox dynamics, and the construction of specific root structural traits.

### 2.4 Cascades of composite injury: from hypoxia-imposed metabolic constraint to whole-plant functional decline

Damage from waterlogging commonly unfolds as a continuous cascade. Rhizosphere hypoxia first suppresses mitochondrial aerobic respiration and sharply reduces ATP supply, imposing immediate energetic limitation on roots. To sustain basal metabolism, plants are forced to intensify glycolysis and activate fermentative compensation to regenerate NAD^+^, however, this strategy is energetically inefficient and frequently accompanied by the accumulation of ethanol, lactate, and related by-products, which further destabilize cellular homeostasis (Chen et al., 2024a). Across genotype comparisons, sensitive lines often exhibit substantially greater fermentative metabolite accumulation than tolerant lines, implying that metabolic maintenance capacity and by-product tolerance may constitute important components of waterlogging tolerance (Gao T. et al., 2025).

As stress persists, energetic constraint, membrane lipid peroxidation, and ionic imbalance jointly drive root hair degeneration, epidermal cell sloughing, and inhibition of root apical growth, leading to a pronounced decline in absorptive function. Simultaneously, excess rhizosphere ions (e.g., Fe^2+^, Mn^2+^, and sulfides) disrupt membrane integrity and depolarize root cells, further impairing nutrient uptake. These root perturbations propagate to the shoot via ABA-, ethylene-, and redox-related signals, altering stomatal behavior and photosynthetic performance, reducing assimilatory capacity and carbon supply, and thereby further eroding the material basis for root renewal and structural remodeling (Zhang R. et al., 2025). Crucially, during post-drainage reoxygenation, the combined impact of rapid respiratory-chain recovery and oxidative pressure from reoxidation of reduced rhizosphere compounds can accelerate ROS accumulation and membrane lipid peroxidation, generating a secondary “reoxygenation oxidative shock” that can, to a considerable extent, dictate recovery rate and the irreversibility of injury (Palma-Silva et al., 2025; Posso et al., 2025).

Collectively, waterlogging injury is not dictated by any single physiological process. Instead, it arises from a coupled set of metabolic and structural consequences initiated by rhizosphere transformation, with severity and reversibility jointly shaped by waterlogging duration, the extent of reductive toxicity, and the rhythm of reoxygenation, among other interacting factors (Rajendran et al., 2025). It is under precisely these constraints that root morphogenetic responses acquire clear adaptive significance. For most cereals (e.g., wheat, maize, barley), survival thresholds typically range from 3 to 7 days of waterlogging at the seedling stage, whereas many legumes (soybean, cucumber) are relatively sensitive and often exhibit severe injury within 2–3 d. Tolerance is jointly determined by metabolic flexibility, aerenchyma formation, ROL barrier deposition, and antioxidant capacity (Ye et al., 2018; Agualongo et al., 2022; Gangana Gowdra et al., 2025).

## 3 Core root morphogenetic modules underpinning waterlogging tolerance: functional differentiation and coordinated timing

Under waterlogging, roots do not respond to hypoxia alone. They face a time-dependent stress environment in which oxygen limitation is followed by energy restriction, accumulation of reduced toxicants, and oxidative injury during reoxygenation (Swift et al., 2025). In this setting, tolerance depends less on the activation of any single pathway than on whether the root system can reorganize its structure quickly enough to protect critical tissues and maintain uptake (Fujita et al., 2021).

**Figure 1:**
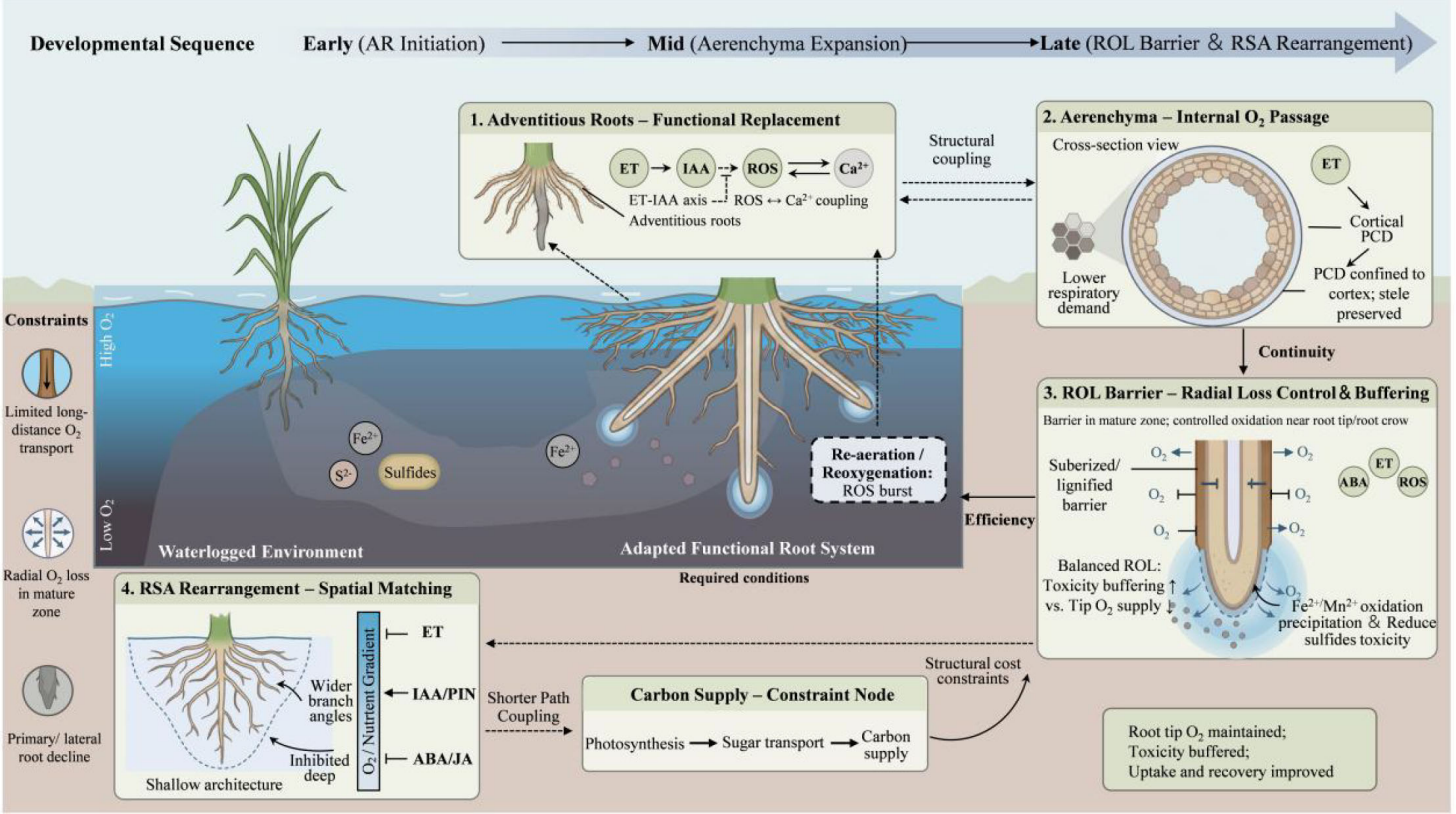
Functional differentiation and phased coordination of four root morphogenetic modules under waterlogging. Waterlogging imposes a temporally structured composite stress characterized by restricted rhizosphere O_2_ diffusion, accumulation of reduced phytotoxins (for example, reduced ions and sulfides), and a ROS burst during reoxygenation after drainage. Under these conditions, plants deploy four linked morphogenetic modules: (1) adventitious root (AR) formation near relatively oxygenated interfaces; (2) aerenchyma development to enhance internal gas diffusion and reduce respiratory cost; (3) formation of a barrier to radial oxygen loss (ROL) in mature root zones to improve oxygen-use efficiency while protecting the root apex; and (4) root system architecture (RSA) remodeling toward shallower soil layers. Solid arrows indicate major processes or temporal progression, dashed arrows indicate conditional coupling or indirect effects, and T-bars indicate inhibition. Abbreviations: AR, adventitious root; RSA, root system architecture; ROL, radial oxygen loss; PCD, programmed cell death; ET, ethylene; IAA, auxin; ABA, abscisic acid; ROS, reactive oxygen species.

These root responses can be understood as coordinated solutions to three major constraints: limited internal oxygen transport, loss of oxygen from mature tissues through ROL, and decline of the pre-existing primary-lateral root system under prolonged hypoxia and chemical toxicity (Huang et al., 2022). Plants therefore tend to deploy four core morphogenetic modules: adventitious root formation, aerenchyma development, ROL barrier formation, and RSA reconfiguration (**Figure 1**) (Kim et al., 2025). These modules are interdependent rather than isolated, and species differences often reflect differences in activation threshold, spatial placement, and module combination (Feng et al., 2023).

### 3.1 Adventitious roots functional root replacement and reconstruction of a near-surface oxidative interface

Under waterlogging, adventitious root emergence is not merely an increase in root number, it functions as a form of “functional root replacement.” When the primary root and its basal laterals experience inhibited aerobic respiration, compromised membrane stability, or reduced apical activity due to hypoxia and the accumulation of reduced phytotoxins, adventitious roots often initiate closer to the soil surface—where oxygen diffusion is comparatively less constrained, thereby establishing a new absorptive root system and providing a structural platform for subsequent aeration and ROL regulation (Chen et al., 2024d; Kim et al., 2018). Consistently, the rate and site of adventitious root formation show an adaptive spatial bias: primordia frequently arise from cell populations at the stem base or hypocotyl near the soil surface. This region benefits from proximity to relatively oxygenated layers and from privileged access to shoot-derived assimilates, satisfying the material and energetic demands of cellular dedifferentiation, primordium initiation, and vascular reconnection (Sha et al., 2024).

Developmentally, adventitious rooting under waterlogging is best viewed as stress-induced organogenesis. Local cell groups re-enter the cell cycle to form primordia, establish a root apical meristem, reconnect to the vascular system, and ultimately generate an independent root unit with its own functional apex (Herzog et al., 2016). Importantly, genotypic differences are typically manifested in initiation timing and functional quality rather than simple root counts: tolerant genotypes tend to initiate primordia earlier and more rapidly establish elongating, persistent apices, whereas sensitive genotypes may produce adventitious roots that nonetheless lose apical activity under sustained hypoxia or reoxygenation-associated oxidative shock (Singh et al., 2023). In horticultural crops, tolerant materials often complete primordium activation within a shorter timeframe, generate a higher proportion of continuously growing adventitious roots, and more readily connect to pre-existing aeration networks at the stem base, thereby improving continuity of internal oxygen transport (Rahman et al., 2023). In wetland or waterlogging-adapted crops, adventitious roots are frequently coupled with enhanced root hair formation and ion uptake capacity, partially compensating for the declining function of the original primary-lateral root system (Owusu et al., 2023).

Mechanistically, adventitious rooting emerges from the convergence of hypoxia signaling and hormonal circuitry. Waterlogging promotes ethylene accumulation, ethylene signaling—via transcriptional regulators such as *EIN3*/*EIL*—interfaces with auxin transport, signaling, and transcriptional responses, creating localized auxin maxima at initiation sites and activating developmental programs involving *ARF*/*LBD* modules to drive primordium formation and vascular reconnection (Gao et al., 2023). In parallel, ROS and Ca^2+^ act as spatiotemporally patterned transducers that influence cell-cycle reactivation and cell-wall remodeling, modulating the activity of expansins and pectin-related enzymes to enable primordium emergence (Gong et al., 2022). Because hypoxia constrains ATP production, progression from “primordium” to “functional root” is often limited by carbon input and sugar mobilization capacity: tolerant genotypes more effectively sustain glycolytic ATP supply and assimilate transport to the stem base/new roots during hypoxia, supporting continued division and differentiation (Ji and Hyun, 2023; Xu et al., 2024).

Notably, the contribution of adventitious roots to waterlogging tolerance depends strongly on downstream structural integration. Without effective aerenchyma continuity or ROL control, adventitious roots may only transiently expand the absorptive interface yet fail to maintain long-term internal oxygenation and apical viability under persistent hypoxia. Thus, evaluation should extend beyond root number to include aeration connectivity, sustained apex vigor, and ion-uptake adaptation (Takahashi et al., 2023).

### 3.2 Aerenchyma building internal oxygen conduits through directed cortical remodeling

Aerenchyma provides the structural foundation for internal oxygen transport under waterlogging, serving two primary functions. First, by establishing a continuous gas-space network, it reduces diffusion resistance and enhances axial oxygen transport from shoot to root apex. Second, by decreasing the proportion of living cortical cells, it lowers maintenance respiration costs, thereby increasing the effective oxygen flux available to the root tip under hypoxia (Martins et al., 2023). Aerenchyma formation is therefore not a simple “appearance of cavities,” but a directed reconfiguration of cortical tissue under energetic limitation: selective loss of cortical cells is traded for higher gas permeability and reduced oxygen demand (Gao T. et al., 2025).

By developmental mode, aerenchyma can be classified as lysigenous or schizogenous. In most crops under waterlogging, lysigenous aerenchyma predominates and depends on programmed cell death (PCD) coupled with cell-wall degradation, whereas some wetland species exhibit schizogenous patterns or mixed characteristics (Zhou et al., 2021). From an ecological/strategic perspective, aerenchyma can also be constitutive or inducible. Constitutive aerenchyma (e.g., in some rice materials) provides a pre-formed gas-space baseline and enables faster responses, whereas inducible aerenchyma (common in many upland crops) is highly plastic but requires rapid establishment of stress signaling and tight spatial control of PCD (Zhou et al., 2025). In waterlogging-sensitive crops, genotypic differences often emerge as differences in induction kinetics, spatial distribution, and network continuity: tolerant materials more consistently generate continuous conduits while sparing vascular tissues and the apical meristem, whereas sensitive materials are more prone to PCD spread and tissue necrosis (Hong et al., 2024).

Mechanistically, ethylene accumulation is widely positioned as a key upstream trigger. Under waterlogging, enhanced ACC synthesis/oxidation and restricted gas diffusion promote ethylene retention, downstream networks (including ERF-family regulators) stimulate ROS production and couple with Ca^2+^ signaling to initiate PCD in specific cortical layers (Zhang R. et al., 2025). Subsequently, cell-wall hydrolases and disassembly factors—such as cellulases, pectinases, and cysteine proteases—are induced to drive selective cortical degradation and gas-space formation. A prevailing view is that the advantage of tolerant genotypes is not “stronger PCD,” but more stringent spatial confinement of PCD—largely restricted to the cortex while avoiding the vasculature and meristem—thereby generating functional aerenchyma without systemic necrosis (Zhang et al., 2024). This spatial restriction likely reflects cell-fate control mechanisms involving antioxidant capacity, autophagy, and the timing of vacuolar rupture, which together tune PCD intensity, boundaries, and duration (Liu et al., 2023).

### 3.3 ROL barrier formation allocating limited oxygen flux and buffering a toxic rhizosphere

Once aerenchyma is established, the limiting factor often shifts from “presence of conduits” to “efficient allocation and utilization of a finite oxygen flux.” ROL is an inherent consequence of axial oxygen transport, ROL barrier formation represents an active strategy to spatially allocate oxygen by reducing non-target leakage from mature zones and thereby increasing oxygen availability at the root tip (Hong et al., 2023). Thus, the ROL barrier should be viewed not merely as a structural feature that “reduces leakage,” but as a regulatory mechanism that balances internal oxygenation against rhizosphere oxidation dynamics (Shiono and Matsuura, 2024).

ROL exhibits a clear duality. Moderate ROL can generate an oxidative microzone around the root surface, promoting oxidation/precipitation of Fe^2+^ and Mn^2+^, reducing sulfide toxicity, and potentially shaping beneficial rhizosphere microbial functions. However, when oxygen flux is limiting, excessive ROL can substantially deprive the apex of oxygen, particularly in severely hypoxic deeper layers, accelerating tip decline (Teoh et al., 2024). Accordingly, many tolerant materials display a spatial pattern characterized by “barrier formation in the mature root zone together with the maintenance of a certain oxidative capacity near the root cap:”: the mature zone barrier minimizes leakage to safeguard apical oxygenation, while the distal region preserves an oxidative interface that helps buffer toxicity (Ejiri et al., 2021).

Histologically, ROL barrier formation is commonly associated with suberization and lignification of cell walls in the exodermis and/or epidermis-associated layers. Under waterlogging, the phenylpropanoid pathway is activated, increased expression of key enzymes (e.g., CCR, COMT) promotes deposition of suberin and lignin, thereby reducing radial gas and solute permeability (Mira et al., 2023). This process is co-regulated by hormones and redox signals: ethylene can promote expression of barrier-associated biosynthetic genes, while ABA modulates deposition intensity and—together with ROS signaling—helps define the spatial boundaries of initiation and expansion (Libao et al., 2024). A major bottleneck in this field is the lack of standardized quantitative descriptors of barrier position, thickness, and chemical composition, which severely limits cross-study and cross-species comparability (Chen et al., 2024a). Establishing direct quantitative linkages among barrier traits, ROL flux, apical oxygen status, and toxic load (e.g., Fe^2+^/S^2-^) would markedly strengthen mechanistic inference (Xie et al., 2025; Chen et al., 2024c).

It is equally important to recognize the physiological costs of barrier formation: suberin/lignin deposition consumes assimilates and can reduce water and nutrient permeability of outer tissues, potentially constraining mineral acquisition (Yang et al., 2023). ROL barrier formation is therefore best conceptualized as a context-dependent strategy rather than a “more is always better” trait. The advantage of tolerant genotypes often lies in the spatial placement and formation rate of the barrier, which are better matched to the dual demands of apical oxygenation and rhizosphere toxicity mitigation (Ateeq et al., 2023).

### 3.4 RSA reconfiguration reshaping root spatial deployment along oxygen-nutrient gradients

RSA reconfiguration represents system-level organization of waterlogging adaptation. In contrast to adventitious roots and aeration structures, which primarily construct oxygen delivery at organ/tissue scales—RSA reshapes root spatial distribution and branching patterns across the soil profile, concentrating root biomass in zones with higher oxygen availability and reprioritizing exploration versus acquisition under oxygen-nutrient heterogeneity (Etame Kossi et al., 2025). Consequently, RSA reconfiguration often manifests as shallow rooting, upward shifts in lateral root initiation sites, increased branching angles, and suppression of deep rooting—features that reduce exposure to severely hypoxic layers (Ye et al., 2018).

This spatial remodeling is not purely passive. Ethylene can inhibit primary root elongation and promote lateral root developmental programs, auxin, via PIN-mediated polar transport, regulates growth direction and branching patterning, ABA and jasmonates contribute to balancing growth with stress responses, shaping the magnitude and persistence of RSA plasticity (Demiss et al., 2025). Waterlogging also alters soil hydraulics and mechanical conditions, mechanosensory and hydraulic signaling pathways may therefore participate in growth-direction and branching decisions, further enhancing RSA responsiveness to environmental gradients (Calabritto et al., 2025). Functionally, shallow rooting can increase survival probability during hypoxia and influence post-drainage recovery; however, potential trade-offs include reduced access to deep nutrient pools and, together with the mechanically weakening effects associated with extensive aerenchyma, an increased lodging risk in some contexts (Devi et al., 2025).

A key mechanistic constraint is carbon supply. Waterlogging often suppresses shoot photosynthesis and perturbs source-sink relationships, while structural construction and maintenance of RSA reconfiguration (as well as adventitious rooting and barrier formation) require sustained assimilate investment. Genotypic differences may therefore partly reflect variation in carbon allocation and sugar metabolic regulation, which sets the upper bound of RSA plasticity (Schweiger et al., 2023). Linking RSA dynamics to internal oxygenation, carbon partitioning, and post-drainage recovery performance will help move the field from “morphological description” toward “functional explanation” (Cardoso et al., 2014).

### 3.5 Module coordination and developmental dependency phased activation and the basis of structural redundancy

The four morphogenetic modules do not contribute via simple additive effects, they form a cooperative network shaped by developmental dependency and physiological constraint. Available evidence supports a broadly consistent phased framework: during early stress, adventitious root primordium initiation and rebuilding of root-type composition often occur first, establishing a developmental substrate for subsequent oxygen-supply structures, aerenchyma then progressively strengthens, constructing axial conduits while reducing cortical oxygen consumption, as stress persists or stabilizes, ROL barrier formation intensifies to improve oxygen-use efficiency, in parallel, RSA reconfiguration finalizes system-level spatial matching to oxygen-nutrient gradients (Zhang X. et al., 2023). This temporal pattern is not invariant: environmental variables, including rhizosphere oxygen status, Eh, and reoxygenation rhythm, can substantially shift activation thresholds and formation rates. Thus, cross-study comparisons should, as far as possible, be conducted under harmonized environmental controls to avoid conflating environmental variation with genetic effects (Shao et al., 2024; Wang et al., 2025).

Studies across genetic materials and treatments further indicate that when one module is constrained, plants may partially compensate by amplifying others. For example, stronger adventitious rooting or a more pronounced upward RSA shift can shorten oxygen transport distance, enhanced aerenchyma can increase axial oxygen delivery, and stronger outer-layer barrier formation can reduce non-target ROL, collectively helping preserve apical oxygenation and baseline absorptive function (Quiñones Martorello et al., 2023). However, not all compensatory combinations are functionally equivalent. Whether compensation translates into a tolerance advantage typically depends on the simultaneous satisfaction of three conditions: continuity of connectivity between root types and aeration structures, maintenance of apical vitality and membrane stability, and alignment between ROL control intensity and the prevailing rhizosphere chemical context (Wasim et al., 2025). Therefore, a more explanatory research trajectory is to analyze “module combinations” jointly with “environmental boundary conditions,” thereby defining which structural packages remain stable and effective under distinct waterlogging scenarios (Hao et al., 2023).

## 4 Cross-Scale regulatory networks: signaling hubs, energy metabolism, and carbon allocation

**Figure 2:**
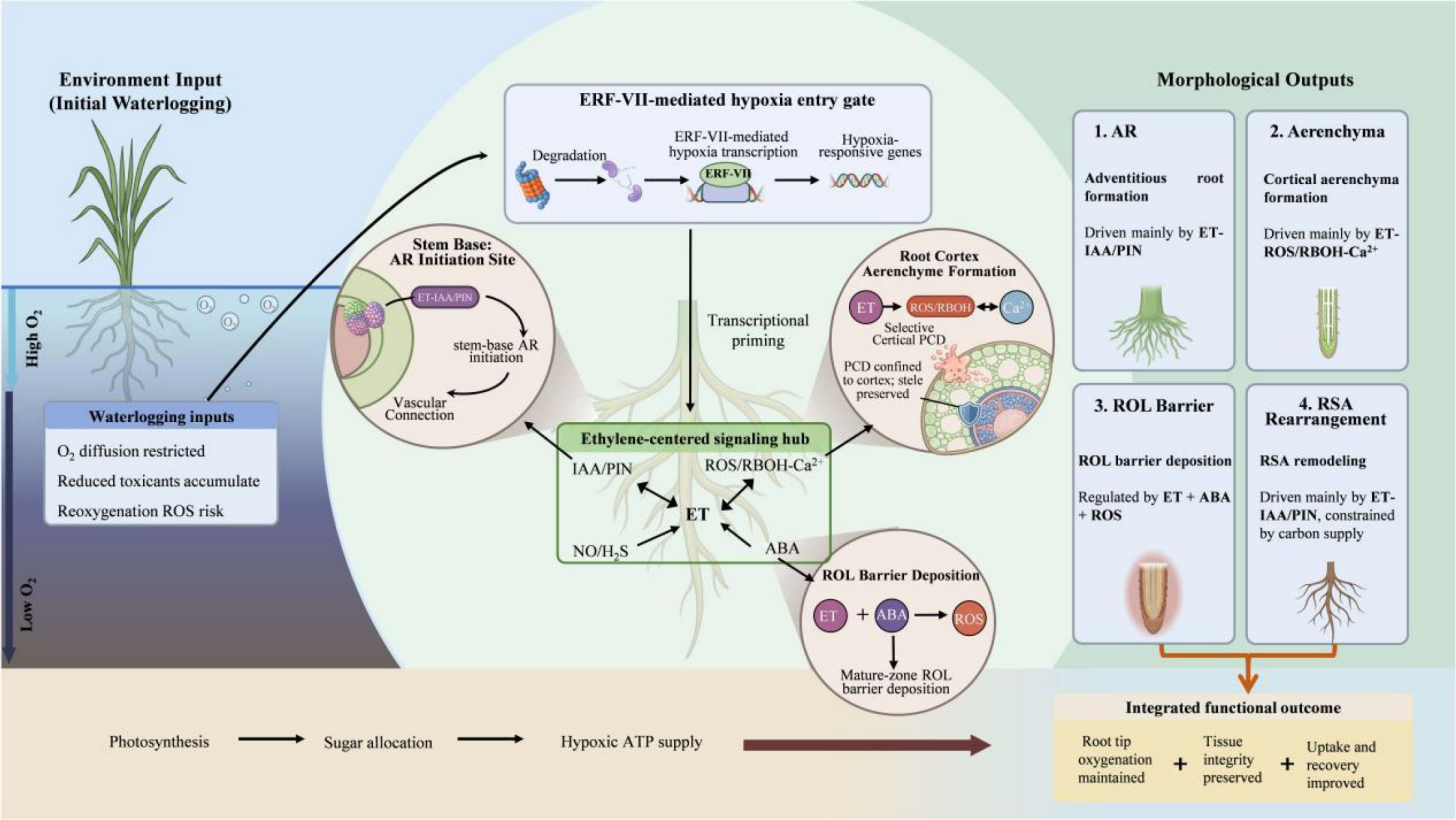
Cross-scale regulatory network of root morphogenesis under waterlogging. Waterlogging causes restricted rhizosphere O_2_ diffusion, a decline in soil redox potential, accumulation of reduced phytotoxins such as Fe^2+^, Mn^2+^, and sulfides. These inputs activate an ERF-VII-mediated hypoxia entry gate and an ethylene-centered signaling hub integrating ethylene-auxin coupling, ROS/RBOH-Ca^2+^ relay signaling, ABA modulation, and gaseous regulators such as NO and H_2_S. The integrated network drives four linked morphogenetic modules: adventitious root formation, cortical aerenchyma formation, ROL barrier deposition, and RSA remodeling. Carbon supply and assimilate reallocation act as a major resource constraint on the construction and maintenance of these structures. Solid arrows indicate primary processes or temporal progression. Abbreviations: ERF-VII, Ethylene Response Factor group VII; RBOH, respiratory burst oxidase homolog; ABA, abscisic acid; NO, nitric oxide; H_2_S, hydrogen sulfide; ROL, radial oxygen loss; RSA, root system architecture; ROS, reactive oxygen species.

Root morphogenesis under waterlogging is not a collateral by-product of stress, it is a concrete manifestation of hypoxia-triggered developmental reprogramming executed within root tissues (**Figure 2**). Current evidence converges on three tightly coupled layers. First, hypoxia sensing and the core transcriptional hypoxia program determine whether a plant enters a *bona fide* “hypoxic mode” and set the sensitivity landscape for downstream morphogenetic outputs. Second, hormonal hubs—most prominently ethylene—together with ROS/Ca^2+^ signaling provide spatially resolved and intensity-tunable control, thereby specifying where and how fast adventitious roots, aerenchyma, and RSA remodeling unfold. Third, the mode of energy supply under oxygen limitation, along with the capacity to mobilize and allocate assimilates, ultimately determines whether these structures can be built and maintained, preserving root apical viability and functional integrity during prolonged stress and through the reoxygenation phase (Eerdekens et al., 2025).

### 4.1 Hypoxia sensing and the hypoxic transcriptional program: the “entry condition” for morphogenesis

Hypoxia is not only a passive consequence of waterlogging. Plants possess a relatively conserved sensing and transcriptional system that allows them to enter a hypoxia-acclimation state. ERF-VII transcription factors are central to this process because their stability changes through oxygen-dependent protein turnover, which in turn reshapes transcription under low oxygen. This regulatory layer determines when the hypoxia program begins and how long it is maintained (Gu et al., 2025). However, hypoxia-responsive transcription alone does not fully determine morphology (Zhang L. et al., 2025). The spatial expression of root morphogenesis still depends on subsequent hormonal integration, redox control, and metabolic support (Laanisto et al., 2025).

### 4.2 Ethylene signaling: an early waterlogging hub that prioritizes morphogenetic programs

Across diverse systems, ethylene-associated signatures repeatedly emerge early during waterlogging and co-occur robustly with adventitious rooting, aerenchyma formation, ROL regulation, and RSA reconfiguration. Collectively, these findings position ethylene not only as an early signal carrier but as a central hub that selects and amplifies morphogenetic programs (Geng et al., 2023; Chen et al., 2024b). Ethylene accumulation reflects both increased ACC biosynthesis/oxidation and restricted gas diffusion following soil inundation, which favors ethylene retention within tissues. Downstream transcriptional outputs mediated by *EIN3*/*EIL* intersect with multiple developmental pathways, enabling ethylene to trigger reprogramming, yet sustained high ethylene can also potentiate senescence-associated processes and elevate injury risk—highlighting a strong intensity- and time-dependence of ethylene action (Li H. et al., 2025; Chen et al., 2024c).

From a morphogenetic standpoint, the association of ethylene with adventitious rooting and RSA remodeling is particularly consistent: ethylene promotes shifts in root-type composition and favors shallow deployment, while its interaction with auxin signaling shapes primordium positioning and branching patterns (Sreeratree et al., 2022). By contrast, aerenchyma formation and ROL barrier development more often reflect ethylene-driven downstream engagement of ROS/PCD modules and wall-deposition programs, whose effective strength is jointly conditioned by tissue layer identity, redox background, and metabolic state (Takahashi et al., 2015).

Importantly, ethylene accumulation is not a “more is better” phenomenon. Waterlogging-tolerant plants often exhibit earlier activation coupled with finer spatial restriction of ethylene signaling—sufficient to induce constructive morphogenesis while minimizing excessive growth inhibition and cumulative tissue damage (Chen et al., 2024a).

### 4.3 Ethylene–auxin crosstalk: a core coupling that converts stress inputs into structural outputs

The capacity of ethylene to drive root morphogenesis largely derives from its ability to reshape auxin distribution and signaling output. Extensive evidence indicates that ethylene modulates the expression and polar localization of auxin transporters (e.g., PIN and ABCB proteins). This modulation re-establishes auxin gradients in the stem base and proximal root regions, converting waterlogging from an environmental input into a spatially explicit developmental signal (Cao et al., 2024). Here, auxin does not simply “promote growth”, through transcriptional modules such as *ARF*/*LBD*, it enables local dedifferentiation, activates adventitious root primordia, and orchestrates integration of newly formed roots into the vascular system, ensuring orderly remodeling within defined tissue domains. This coupling displays pronounced spatial selectivity: adventitious roots preferentially form near relatively oxygenated interfaces and in regions with stronger carbon supply, maximizing functional returns under hypoxia (Yan et al., 2024). Work in horticultural crops (e.g., cucumber) provides a particularly coherent chain of evidence: rapid adventitious rooting aligns with synchronous ethylene/auxin dynamics and tracks varietal differences in waterlogging tolerance, offering an actionable framework to trace “phenotypic divergence” back to discrete regulatory bottlenecks (Fagerstedt et al., 2024).

Overall, the ethylene-auxin axis not only determines the direction and magnitude of adventitious rooting and RSA remodeling, but also reshapes root-type composition and spatial deployment, thereby laying the structural groundwork for where aerenchyma formation and ROL control can be most effectively established (Wang et al., 2024).

### 4.4 ROS/RBOH–Ca^2+^ signaling: redox control of cell fate and tissue-layer positioning

Redox signaling—particularly the production and regulation of ROS—constitutes a central mechanism through which plants execute morphogenetic remodeling under waterlogging. ROS are well recognized as major contributors to cellular injury during reoxygenation, however, under low O_2_ they also function as *bona fide* signaling molecules that impose temporal and spatial control over morphogenetic programs (Zhong et al., 2023). ROS production is commonly driven by RBOH enzymes, which can be activated under hypoxic stress and operate in concert with Ca^2+^ pathways, forming a spatiotemporally gated relay network (Nakamura et al., 2025). ROS are pivotal for aerenchyma development, where they help initiate and pattern cortical PCD to generate structural gas spaces (Zhu et al., 2025). During adventitious rooting and RSA remodeling, ROS can also shift thresholds for cell division and elongation, influencing primordium initiation and vascular reconnection dynamics (Bianucci et al., 2025).

Ca^2+^ acts as an essential second messenger that closely interlocks with ROS signaling to shape cell-fate decisions. By modulating cytoskeletal dynamics, membrane stability, and ROS-dependent signaling outputs, Ca^2+^ signaling can promote membrane integrity and antioxidant enzyme activity, thereby enhancing tolerance to oxidative stress during both hypoxia and reoxygenation (Di Bella et al., 2026). The ROS-Ca^2+^ interplay therefore provides a key spatiotemporal control platform that enables precise structural remodeling in response to the coupled challenges of oxygen limitation and reoxygenation-driven oxidative pressure.

### 4.5 Gaseous signals: tuning redox thresholds and intersecting with hormonal networks

In waterlogging responses, gaseous signals such as NO and H_2_S contribute not only to antioxidant defenses but also to morphogenetic control. NO can modulate antioxidant capacity and hormonal outputs, thereby fine-tuning ROS production and the extent of cellular damage and, in turn, shifting the “activation conditions” for morphogenetic responses (Asghar et al., 2025). Through crosstalk with ethylene and ABA networks, NO influences ROS peak formation, helps stabilize membranes under stress, and can promote adaptive responses when damage accumulates (El-Khateeb et al., 2023). Beyond antioxidation, NO may also shape the dynamic balance between internal oxygen delivery and rhizosphere oxidative microzones, thereby affecting apical oxygenation and, ultimately, root growth and uptake function (Bittencourt et al., 2023).

Although less frequently emphasized, H_2_S appears to play key roles in sustaining mitochondrial metabolic homeostasis and antioxidant defenses under waterlogging (Tyagi et al., 2022). Similar to NO, H_2_S can help maintain metabolic activity across extreme hypoxia and subsequent reoxygenation by tuning redox balance. The interaction between NO and H_2_S may optimize redox homeostasis and, via broader signal integration, enhance tolerance to waterlogging stress (Nguyen et al., 2018).

Together, NO and H_2_S provide a modulatory layer for redox-state control. In morphogenesis, this layer likely contributes to the precise timing and spatial delimitation of cell division, elongation, and vascular reconnection. While mechanistic details remain complex, the dynamic balance of gaseous signals is evidently consequential for root adaptation to waterlogging environments.

### 4.6 Energy-metabolic reprogramming and carbon allocation: the energetic foundation and resource constraints of morphogenesis

Morphogenetic construction—primordium activation, cell division and elongation, cell-wall remodeling, and suberin/lignin deposition—requires sustained energy and carbon inputs. Thus, waterlogging-tolerant morphogenesis depends not only on “signal initiation” but on whether plants can maintain energy supply and material provisioning under low O_2_ (Jia et al., 2024). The frequent co-occurrence of anaerobic metabolism/fermentation with multiple morphogenetic modules suggests that metabolic reprogramming and structural construction are often synchronized. Under hypoxia, glycolysis and fermentative compensation become dominant routes to sustain ATP generation, whereas alternative respiratory pathways (e.g., the alternative oxidase (AOX) route) may mitigate over-reduction of the electron transport chain and reduce ROS risk, supporting survival and post-waterlogging recovery (Nguyen et al., 2025; Wu et al., 2024).

Carbon allocation imposes an additional constraint. Waterlogging commonly suppresses shoot photosynthesis and perturbs source-sink relationships, leading to inadequate carbon supply. Tolerant genotypes often maintain higher glycolytic capacity during hypoxia and more effectively divert assimilates to the stem base and newly formed roots, supporting sustained division and differentiation. In contrast, sensitive genotypes exhibit poorer carbon allocation, restricting both the magnitude and durability of structural remodeling (Secomandi et al., 2025).

From an integrative perspective, energy supply and carbon availability directly determine whether structural traits can be built efficiently and maintained, thereby shaping adaptive performance under waterlogging. Mechanistic resolution will be strengthened by explicitly linking metabolic indices (e.g., ATP/ADP ratio and glycolytic activity) with morphogenetic traits (adventitious rooting, aerenchyma establishment, ROL barrier strengthening) and functional readouts (apical oxygen status, ROL flux, and post-drainage recovery), enabling the field to progress from “morphological description” to “functional explanation” (Alghamdi et al., 2025).

## 5 Interspecific and genotypic divergence: ecological adaptation of root morphological strategies

Differences in waterlogging tolerance across species and genotypes fundamentally arise from “species-/genotype-specific spatiotemporal diversification” in how key morphogenetic modules—adventitious rooting, aerenchyma formation, ROL barrier construction, and RSA remodeling, are combined, phased, and regulated under distinct waterlogging regimes. These divergence patterns represent the evolutionary outcome of long-term adaptation to “hydric (wetland) versus mesic/upland niches” and to differences in stress intensity, duration, and rhythmicity. At the molecular level, they are increasingly attributable to lineage- and genotype-specific differentiation in “hypoxia-pathway sensitivity, carbon-metabolic efficiency, and gene-expression programs” (Langan et al., 2024). Notably, classifying waterlogging by “severity, persistence, and reoxygenation dynamics” provides a more precise framework for dissecting the mechanistic basis of adaptive morphogenetic strategies.

### 5.1 Divergent patterns of waterlogging-adaptive root morphogenesis across species

Root morphogenetic responses to waterlogging are strongly “species dependent”, reflecting habitat history, ecological constraints, and evolutionary trajectories. There is no universal “waterlogging morphology.” Instead, strategies diversify in accordance with the “magnitude and volatility of habitat water regimes”, revealing multiple viable solutions at physiological, ecological, and evolutionary levels and underscoring the breadth of adaptive root remodeling under contrasting stress contexts (Sugiura et al., 2024).

Species associated with habitats that undergo long-term or frequent inundation generally prioritize traits that maximize internal aeration efficiency. Wetland-adapted species belong to this broad ecological group and typically include plants from permanently wet or regularly flooded environments. In these species, dense adventitious roots, well-developed aerenchyma, and robust ROL barriers often work together to support root metabolism during sustained hypoxia (Cheng et al., 2015). By contrast, mesic or upland species more often rely on strategies that reduce respiratory burden, shorten the effective exposure window, or redistribute roots toward soil layers with higher oxygen availability (Ge et al., 2024). This distinction clarifies that “wetland-adapted” species are not a separate category from plants of periodically inundated or permanently wet habitats, but rather represent a major adaptive subset within that habitat spectrum.

Interspecific variation in RSA remodeling is equally consequential. Wetland species frequently exhibit pronounced upward root relocation (shallow rooting) to enhance access to oxygenated soil layers, whereas upland species may reduce branching density to curb respiratory costs. Because waterlogged soils display sharp spatial heterogeneity in oxygen and nutrient availability, plants can improve performance by reshaping the spatial distribution of roots to exploit relatively oxygen-rich zones and to optimize the oxygen-nutrient trade-off (Zhang B. et al., 2023). These patterns reflect adaptive prioritization of resource allocation under waterlogging-imposed constraints.

Critically, interspecific (and inter-genotypic) divergence in tolerance often manifests as differences in module activation thresholds, response magnitude, timing control, and combinatorial assembly of morphogenetic traits. In effect, plants optimize hypoxia adaptation by deploying diverse, niche-tuned “module packages,” achieving distinct but locally optimal solutions under low-oxygen stress (**Table 1**) (Olorunwa et al., 2022).

**Table 1:** Cross-species variation in root morphological modules conferring waterlogging tolerance and associated ecological adaptive traits under waterlogging stress.

Ecological habitat	Species	Module assemblage	Key indicators	Primary benefits and trade-offs	References
Long-term or high-frequency inundation (typical wetland habitats)	Rice, *Phragmites*, wetland grasses, etc.	AR↑; aerenchyma (constitutive/rapidly inducible)↑; mature-zone ROL barrier↑; RSA shallowing↑	Rate of AR formation and proportion of sustained elongation; root porosity/aerenchyma continuity; mature-zone ROL flux/barrier diffusion resistance; shallow-root fraction and root angle.	Maintains oxygen supply to the root apex, buffers Fe^2+^/S^2−^ phytotoxicity, and stabilizes rhizosphere microenvironments. Trade-offs: higher carbon cost and weaker access to deep resources.	(Kim et al., 2025; Jia et al., 2024; Nakamura et al., 2025)
Hypoxia compounded by reductive toxicity (intertidal zones/salt marshes/wetland shrubs)	Salt-marsh shrubs; coastal plants (e.g., *Suaeda* spp.); mangroves, etc.	ROL barrier ↑; aerenchyma ↑; AR and/or respiratory structures ↑; near-surface RSA ↑	Suberin/lignin deposition; rhizosphere Eh; oxidative microzone scale; root-tip survival.	Limits non-target oxygen loss and reduces chemical injury. Trade-offs: Reduced water/nutrient uptake; high tissue construction costs.	(Dey et al., 2025; Asghar et al., 2025; Cheng et al., 2015)
Seasonal waterlogging/short- to mid-term waterlogging (upland cereals)	Maize, wheat, barley, and other cereals	Nodal roots or AR↑; inducible aerenchyma↑; conditional ROL barrier; shallow RSA↑.	Threshold and time window for nodal root/AR initiation; aerenchyma continuity and boundaries of programmed cell death; ROL distribution; root-depth shift.	Rapid rebuilding within a limited low-O_2_ window. Trade-offs: carbon investment and lodging risk.	(Devi et al., 2025; Zhu et al., 2025; Gao H. et al., 2025)
Sporadic or sudden waterlogging (mesophytic vegetables/legumes)	Soybean, cucumber, tomato, etc.	AR; weak aerenchyma; weak or slow-forming ROL barrier; shallow RSA or lateral-root suppression	AR-aeration connectivity; root-apex viability and membrane stability; root carbon reserves and respiratory consumption; regrowth capacity.	Supports short-term uptake with near-surface roots. Trade-offs: insufficient internal aeration and barrier protection; energy crisis and oxidative shock.	(Kim et al., 2018; Eerdekens et al., 2025; Li G. et al., 2025; Wen et al., 2024)
Model plants/mechanistic systems (limited ecological adaptation)	Arabidopsis thaliana and other model species	Growth inhibition, RSA adjustment; aerenchyma and ROL barrier not prominent.	ERF-VII threshold; root growth rate; upward shift of lateral root initiation sites; reoxygenation ROS indices and root-apex survival.	Facilitates dissection of signaling hubs and activation thresholds. Trade-offs: structural adaptations.	(Teoh et al., 2024; Mira et al., 2023)

### 5.2 Intraspecific genotypic divergence: plasticity boundaries of morphogenetic modules

Beyond interspecific differentiation, substantial genotypic variation exists within the same species in root morphogenetic responses under waterlogging. Such variation most commonly reflects differences in activation thresholds, response intensity, and maintenance capacity of existing modules rather than the emergence of entirely new structural types (Kim et al., 2025). Genetically, waterlogging tolerance therefore often represents tunable control over conserved developmental programs, not morphological invention.

A large body of evidence indicates that tolerant genotypes can initiate adventitious rooting and/or aerenchyma formation under lower oxygen partial pressure and can preserve functional integrity of these structures during prolonged stress (Ullah et al., 2024). In sensitive genotypes, induction signals may be detectable, yet structural formation is frequently delayed, limited in scale, and more readily aborted under energy constraint. Such patterns are consistently observed in breeding materials across crops, including maize, wheat, and cucumber, where adventitious rooting rate, cortical porosity, and ROL regulation show stable cultivar-to-cultivar variation (**Table 2**) (Gulzar et al., 2024).

**Table 2:** Representative intraspecific genotypic contrasts in root morphogenetic modules and regulatory features under waterlogging.

Species/genotype	Module assemblages and temporal dynamics	Metabolic and carbon-support differences	Functional outputs and transferable indicators	References
Sesame (*Sesamum indicum*) (tolerant vs sensitive)	Tolerant lines: AR earlier ↑; aerenchyma continuity ↑; mature-zone ROL barrier ↑; shallow RSA ↑.	Tolerant lines: Fermentation stability ↑; carbon allocation to new roots ↑; antioxidant capacity ↑.	Timing of AR initiation; intensity of ROL-barrier deposition, post-drainage new-root recovery rate.	(Hong et al., 2023; Ge et al., 2024; Cao et al., 2025)
Oilseed rape (*Brassica napus*) (tolerant vs sensitive)	Tolerant lines: AR-aerenchyma coordination ↑; ROL barrier ↑; RSA decline weaker.	Tolerant lines: Energy supply stability ↑; favorable assimilate partitioning; antioxidant enzyme activities↑; lipid peroxidation↓.	ROL-barrier deposition strength; carbon sink strength of new roots, MDA, antioxidant enzyme activities.	(Ullah et al., 2024; Mira et al., 2023; Ambros et al., 2022)
Cucumber (*Cucumis sativus*) (tolerant vs sensitive)	Tolerant lines: AR-aerenchyma functional coupling↑; aerenchyma continuity↑, ROL barrier↑, upward shift of RSA↑.	Tolerant lines: Fermentation burden↓; carbon priority to new roots ↑; antioxidant persistence ↑.	AR–aerenchyma connectivity, porosity, ROL-barrier deposition, recovery rate of root viability after reoxygenation.	(Pan et al., 2024a, 2024b)
Rice (*Oryza sativa*) (tolerant vs sensitive)	Tolerant lines: Constitutive/rapid aerenchyma ↑; early ROL barrier ↑; AR integration ↑; stable shallow RSA ↑.	Tolerant lines: Assimilate retention to stem base/new roots ↑; compensatory glycolysis maintained.	Survival rate and biomass; root porosity and root-apex O_2_ status, mature-zone ROL flux, root-apex viability and post-drainage recovery, yield loss.	(Kim et al., 2025; Nguyen et al., 2025)
Maize (*Zea mays*) (tolerant vs sensitive)	Tolerant lines: Nodal roots/AR earlier↑; clearer PCD boundaries; conditional barrier↑; RSA upward shift ↑.	Tolerant lines: Sugar reserve ↑; source-sink reallocation ↑; energy-crisis cascade delayed.	Nodal-root survival; root porosity, root-apex viability, root-depth distribution, post-reoxygenation growth recovery, field lodging and yield stability.	(Feng et al., 2023; Rahman et al., 2023; Xie et al., 2025; Devi et al., 2025)
Barley (*Hordeum vulgare*) (tolerant vs sensitive)	Tolerant lines: Inducible aerenchyma + AR coordinated ↑; localized ROL barrier ↑; shallow RSA aligned with uptake.	Tolerant lines: Sugar-metabolism/antioxidant coordination ↑; respiratory recovery↑.	Root porosity/ROL measurements, spatial boundaries of PCD, root-apex oxygen status; net growth rate during recovery and yield components.	(Sherwood et al., 2025; Shiono and Matsuura, 2024; Zhu et al., 2025; Langan et al., 2024; Zhu et al., 2024)
Soybean (*Glycine max* L.) (tolerant vs sensitive)	Tolerant lines: AR formation ↑; aerenchyma ↑; ROL barrier induction ↑; shallow RSA ↑.	Tolerant lines: Higher fermentation efficiency; enhanced antioxidant capacity; better ionic balance.	AR numbers; aerenchyma porosity; ROL barrier intensity; survival rate after waterlogging.	(Gangana Gowdra et al., 2025; Agualongo et al., 2022)

Importantly, genotypic divergence rarely reduces to a univariate “strong versus weak” contrast, it is more accurately described as module-combination specificity. Some materials excel in adventitious root production but exhibit limited aerenchyma development or ROL control, whereas others improve oxygen-use efficiency primarily by strengthening barrier formation (Lian et al., 2023). Thus, explaining tolerance differences requires focusing on how module packages match rhizosphere boundary conditions, rather than evaluating any single trait in isolation.

### 5.3 Coordinated construction of adventitious roots and aeration tissues: a functional hub of tolerance

Under waterlogging, adventitious root formation and aerenchyma development are not independent events. Their “tight coordination” is a defining functional feature of tolerant root systems, jointly constituting the core unit for oxygen acquisition and internal transport. In tolerant genotypes, adventitious root primordia are commonly activated rapidly and quickly establish continuous connectivity with stem-base gas-space networks. Concomitantly, the frequency of aeration-structure induction within the cortex of newly formed roots is substantially higher than in sensitive materials (Zhu et al., 2024). This “structural coupling” enables new roots to simultaneously act as absorptive organs and as optimized aeration modules (enhanced transport with reduced respiratory burden).

Developmentally, coordination reflects not merely temporal overlap but shared upstream signaling environments and downstream functional integration. Local ethylene accumulation at the stem base can both (i) reshape auxin distribution to promote adventitious root initiation and (ii) activate ROS-linked pathways that induce cortical PCD, laying the anatomical basis for gas-space formation (Chugh et al., 2024). Here, spatial restriction of ROS is decisive: tolerant materials often confine cell death to specific cortical layers, avoiding vascular damage and thereby sustaining long-term functionality of the newly established roots (Wen et al., 2024).

Importantly, increased adventitious root number does not automatically translate into tolerance advantage, functional efficacy depends strongly on synchronized aeration configuration. Comparative studies consistently show that abundant adventitious roots without sufficient aerenchyma still leave root apices at high hypoxia risk, limiting growth maintenance (Shikha et al., 2024). From a functional perspective, adventitious roots should therefore be conceptualized as carriers for aeration function, rather than as an isolated tolerance trait—an interpretive shift that helps explain both interspecific and genotypic divergence.

### 5.4 ROL barrier formation and regulation: balancing internal oxygen supply with rhizosphere detoxification

As aerenchyma develops and internal oxygen transport improves, ROL becomes a key determinant of functional efficiency. Tolerant plants do not simply suppress oxygen leakage. Instead, they build ROL barriers in defined root regions to achieve spatially precise oxygen allocation (Yamauchi et al., 2019), reducing non-productive oxygen loss from mature zones while preserving oxygen delivery to the root tip and lateral-root initiation domains.

Structurally, ROL barriers are primarily based on suberin and lignin deposition in exodermal/endodermal cell walls, typically accompanied by sustained upregulation of phenylpropanoid-pathway genes (Li H. et al., 2025). Unlike adventitious roots and gas spaces, barrier establishment often occurs later, consistent with a role in “optimizing the efficiency” of an already formed aeration system rather than initiating the earliest stress response (Peralta Ogorek et al., 2021).

Regulatorily, barrier formation is shaped by coordinated ethylene, ABA, and ROS signaling. Ethylene promotes expression of suberin-biosynthesis programs in outer tissues, whereas ABA may restrain excessive deposition to preserve necessary permeability (Hu et al., 2022). ROS participate as triggers whose magnitude and duration define tissue specificity of deposition (Sharmin et al., 2024). Here, “rhizosphere oxidative detoxification” refers to the localized oxidation or precipitation of reduced toxicants around the root surface when limited oxygen leaks into the surrounding soil. This process can help convert potentially harmful compounds such as Fe^2+^, Mn^2+^, and sulfides into less harmful forms, thereby buffering chemical injury near sensitive root tissues. In tolerant genotypes, reduced mature-zone ROL combined with controlled oxygen leakage near the root tip helps balance internal oxygen conservation with local chemical protection (Pawar et al., 2025).

### 5.5 Genotypic divergence: coupled constraints of developmental plasticity and metabolic carrying capacity

Beyond local structural traits, waterlogging reshapes RSA at the system level, determining how roots spatially adapt to oxygen distribution and nutrient availability. A broad consensus indicates that waterlogging suppresses primary-root elongation while increasing the proportion of laterals and fine roots in near-surface soil, producing a characteristic shallow root architecture (Thapa et al., 2025). This redistribution reduces exposure to deeper soil layers with more severe oxygen limitation or stronger reducing conditions and shortens the axial distance for oxygen transport from shoots to root apices.

RSA remodeling does not occur independently of adventitious rooting, aerenchyma formation, or ROL barriers, rather, these processes share hormonal and metabolic contexts and thus form an integrated response. Adventitious roots directly reconfigure root-type composition, supplying new structural units for RSA reorganization, aerenchyma lowers respiration cost per unit root length, enabling shallow roots to retain higher functional capacity under hypoxia (Liang et al., 2025). The oxygen-allocation effects of ROL barriers can further amplify the relative advantage of shallow, newly formed roots.

**Figure 3:**
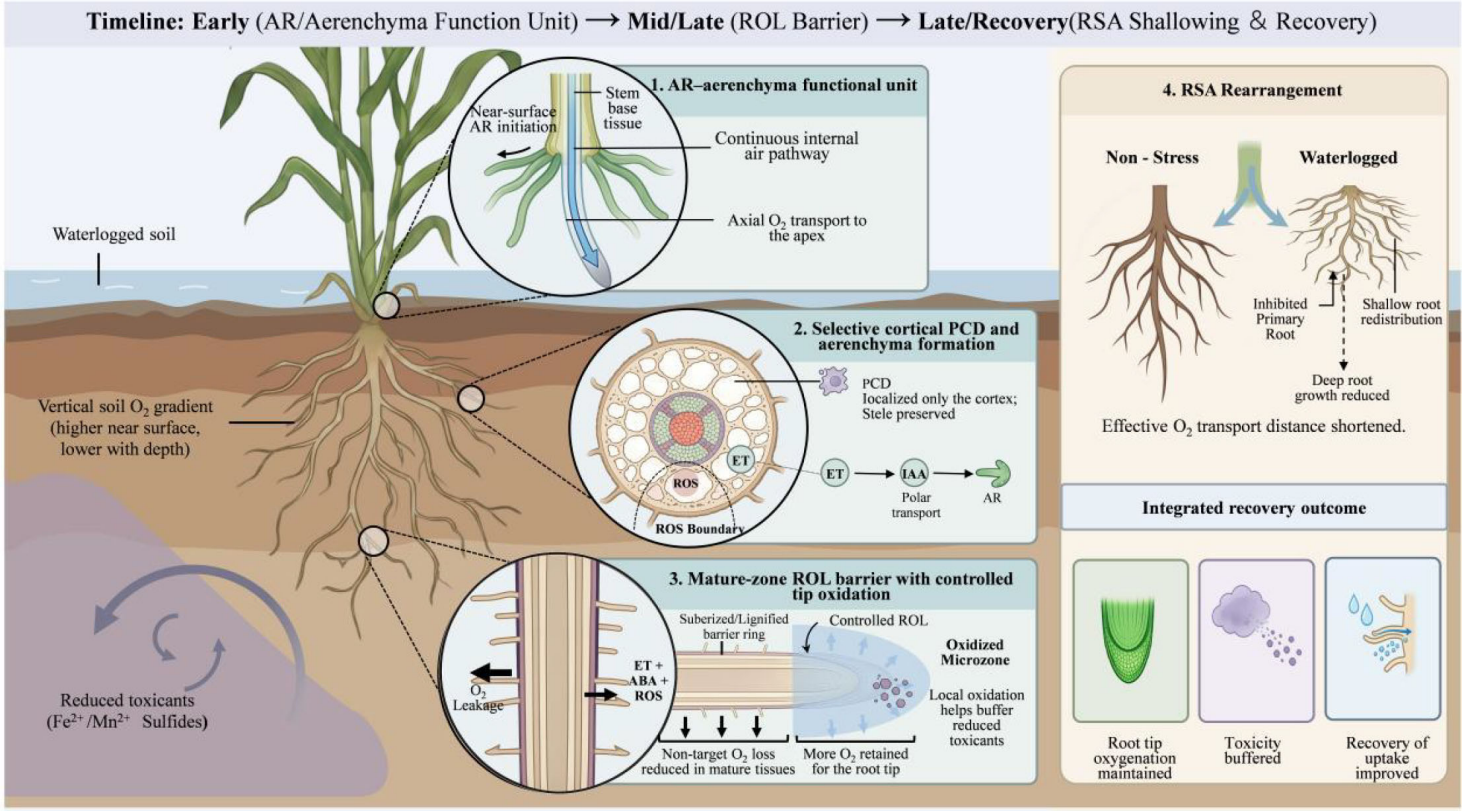
Integrated regulation of coordinated root morphogenetic modules under waterlogging: synergy between adventitious roots and aerenchyma, spatially precise ROL barrier control, and RSA remodeling as a unified adaptive program. Waterlogging imposes a temporally structured stress characterized by rapid decline in soil oxygen after inundation, accumulation of reduced toxicants, and oxidative challenge during post-drainage reoxygenation. In the early phase, adventitious roots (1) formed near the stem base are functionally coupled with aerenchyma development, (2) generating a root unit that supports both uptake and internal aeration. In the mid/late phase, ROL barrier formation (3) reduces non-productive oxygen leakage from mature zones while maintaining controlled oxygen release near the root tip for local chemical buffering. In the late and recovery phase, RSA remodeling (4) shifts root deployment toward shallower and denser architectures dominated by newly formed roots, thereby shortening effective oxygen-transport distance and accelerating functional recovery after drainage. The left-side environmental label has been clarified as “soil oxygen gradient after waterlogging.” Arrows indicate O_2_ transport, ROS signaling, causal activation, integration, or inhibition, as indicated. Abbreviations: AR, adventitious root; ROL, radial oxygen loss; RSA, root system architecture; ROS, reactive oxygen species.

Notably, the adaptive significance of RSA remodeling extends beyond survival during hypoxia: it is also pivotal for post-drainage recovery. Evidence suggests that genotypes with stronger shallow rooting and higher adventitious-root fractions often show faster recovery of hydraulic conductance and nutrient uptake upon reoxygenation, consistent with high activity of newly formed root tissues and reduced respiratory burden conferred by aeration structures (Combs-Giroir et al., 2024). From a dynamic-adaptation perspective, RSA remodeling is therefore better viewed as a bridge between “stress acclimation” and “recovery growth”, rather than a simple avoidance strategy (**Figure 3**).

## 6 Conclusions and outlook

Waterlogging is a major constraint on plant growth and yield, and adaptive remodeling under waterlogging represents a complex, system-level response rather than an isolated stress symptom. To withstand waterlogging, roots deploy a coordinated suite of morphogenetic modules—adventitious root formation, aerenchyma development, establishment of a ROL barrier, and reconfiguration of RSA. These modules do not operate independently, instead, they are integrated through hormonal control and metabolic crosstalk, enabling plants to maintain root function across both the hypoxic phase and the subsequent reoxygenation period. In this review, we synthesized current understanding of the molecular mechanisms, regulatory networks, and ecological logic that underpin root morphogenetic adaptation to waterlogging. By comparing strategies across species and genotypes, we highlighted the species-specificity of root responses—particularly the timing and spatial deployment of distinct modules under low oxygen. The observed divergence is tightly coupled to rhizosphere dynamics and also reflects differences in resource allocation priorities and metabolic efficiency under stress. Notably, hypoxia tolerance thresholds differ markedly across species: cereals generally tolerate 3–7 days of waterlogging at the seedling stage, while legumes are often injured within 2–3 days. Tolerance arises from the coordinated effects of metabolic reprogramming, aerenchyma formation, ROL barrier establishment, and RSA remodeling, rather than any single mechanism.

Despite substantial progress, several important gaps remain. First, many studies still lack standardized descriptors of waterlogging intensity, duration, and reoxygenation regime, which limits direct comparison across species and genotypes. Second, mechanistic work remains too often module-centered, focusing on a single trait or pathway without adequately resolving how traits interact, trade off, or reinforce one another. Future research should therefore adopt a systems perspective and examine how morphogenetic modules are coordinated in space and time to sustain root function and support recovery in a chemically dynamic rhizosphere. Equally important, the field still needs stronger links between mechanism and application. Translating knowledge of root morphogenesis into breeding targets and management strategies remains a central task for climate-resilient agriculture. This review clarifies the cross-scale links among morphological modules, regulatory networks, and environmental matching. It also identifies leverage points for genetic improvement and provides a conceptual basis for molecular breeding and waterlogging-resilient cultivation practices.

Looking forward, progress will depend on deeper coupling between mechanistic resolution and production-oriented deployment. Interdisciplinary innovation—spanning phenomics, genetics, rhizosphere chemistry and microbiome science, and ecophysiology—will be required to translate root morphogenetic principles into measurable selection indices and implementable management protocols. Ultimately, such integration should strengthen yield stability under increasingly frequent waterlogging events and provide both theoretical grounding and practical guidance for sustainable agricultural development.
